# Web-Based Group Photovoice Through the Lens of Survivors of Critical Illness Recovery: Photovoice Qualitative Pilot Study

**DOI:** 10.2196/66601

**Published:** 2026-01-20

**Authors:** A Fuchsia Howard, Kelsey Lynch, Anita David, Rinila Haridas, Leanne M Currie, Sally Thorne, Gregory Haljan

**Affiliations:** 1 School of Nursing The University of British Columbia Vancouver, BC Canada; 2 Faculty of Medicine The University of British Columbia Vancouver, BC Canada; 3 Fraser Health Surrey, BC Canada

**Keywords:** critical illness, recovery, post-intensive care, survivor, photovoice, qualitative, patient engagement, survivors, web-based, pilot study, illness, wellness, photograph discussions, survivor-generated photographs, feasibility, individual interviews, interviews, web-application, therapeutic

## Abstract

**Background:**

The broad spectrum of issues that survivors face after critical illness and the contextual factors that help or hinder remain underexplored, as do their perspectives on what is important during recovery. Photovoice methods offer a means to convey experiences through participant-generated photographs and related narratives that can extend existing notions of illness and wellness.

**Objective:**

This pilot study aimed to (1) describe intensive care unit (ICU) survivor recovery after hospital discharge depicted through survivor-generated photographs and photograph discussions, (2) assess the feasibility of recruiting and retaining participants in a web-based group photovoice focused on ICU recovery, and (3) describe the impact of study participation.

**Methods:**

The web-based group photovoice involved 5 weekly 2-hour group discussion sessions, after which individual interviews were conducted with each participant. Photographs and transcript data from the group discussions and individual interviews were analyzed using a qualitative interpretive description approach.

**Results:**

A total of 5 ICU survivors (4 women and 1 man) participated. The aspects of critical illness recovery that featured prominently included (1) protracted physical recovery; (2) profound psychosocial challenges encompassing fear of the future, emotional turmoil, shifting self-perception, changes in family dynamics, and feelings of disconnection; (3) discrepancies in health needs and support offered; and (4) need for perseverance and resilience. The web-based group photovoice was feasible, with participation characterized as an opportunity to build social connections and draw strength from fellow survivors of critical illness unavailable elsewhere.

**Conclusions:**

Given the compelling insights our pilot study provided into lesser-explored aspects of critical illness recovery, along with its potential therapeutic value and ability to foster social connectedness, future research is warranted to assess the impact of a scaled-up application.

## Introduction

There has been a shift in critical care from a focus on survival alone to considerations of quality of life and longer-term recovery. The large majority of people with a life-threatening illness that is treated in an intensive care unit (ICU) will now survive. Among these survivors, more than half experience postintensive care syndrome (PICS)—a myriad of physical, emotional, and cognitive challenges which often co-occur [1,2]. Physical impairment can include muscle weakness, pain, and fatigue, while cognitive impairment can manifest as impaired memory, poor executive function, and inattention [1,3-5]. The most commonly reported psychological challenges include anxiety, depression, and symptoms of posttraumatic stress disorder [3,4]. PICS can endure long-term, although most research has focused on the first 2 years after hospital discharge [6,7]. As a consequence of PICS, survivors commonly experience difficulties with daily life and poor health-related quality of life [8]. Health care needs can also be substantial, with pooled data indicating that 38%-65% of survivors of critical care are readmitted to the hospital in the first year after critical care discharge [9].

Research over the past decade has yielded robust evidence regarding PICS. However, much of this evidence has been focused on a relatively narrow range of medical challenges deemed important by researchers, overlooking the priorities identified by survivors themselves. Current PICS research has described challenges to self-identity, existential worries, disrupted family and interpersonal relationships, financial difficulties, and employment challenges [1,3]. Yet, the broad spectrum of issues that each survivor faces after critical illness and the contextual factors that help or hinder their experiences remain underexplored. Further, only limited research has identified survivors’ perspectives on what is important. Moreover, while the development and dissemination of information that describes PICS is necessary, it alone is likely insufficient to fully support survivors and their families. There is a pressing need to explore effective ways to communicate the broader impact of critical illness. This includes extending the focus beyond mere medical issues to address the myriad ways in which life is altered and the diverse experiences of those affected. Efforts to complement medically focused education with innovative communication and engagement strategies could greatly enhance support for survivors. Arts-based research, including web-based group photovoice, offers an innovative, participatory means of illuminating the diverse, underexplored experiences and perspectives of ICU survivors and communicating the broader personal and social impacts of critical illness beyond medical PICS outcomes, thereby informing more meaningful and supportive post-ICU care.

Photovoice methods, conceived by Wang and Burris [10], are arts-based and grounded in the tenets of participatory action research, where community members are empowered to capture and share their health and illness experiences using photographs and narratives. Photovoice provides a process through which participants create a visual, metaphoric representation of illness, facilitating the production of rich, nuanced narratives that convey their experiences, which can extend existing notions of illness and wellness [11]. Photovoice data goes beyond verbal interviews and text alone and is an excellent means for distilling nuanced, emotional, internal, and subjective experiences [12]. Additionally, photovoice empowers participants to not only document but also actively shape, interpret, and articulate what matters to them in their health and illness narratives. Moreover, Photovoice can generate portrayals of health challenges that complement medical information by conveying complex and diverse experiences from patients’ perspectives [13]. Viewing authentic experiential portrayals may help people glean insight, hope, and validation, and feel they are not alone [14]. Photovoice, in particular, has the potential to raise awareness of issues that matter to communities as a step to advocacy leading to social change [15].

Using a longitudinal qualitative design, our team has been investigating critical illness recovery at home and drivers of hospital readmission through patient and family caregiver perspective evidence. Recruitment and data collection for the larger project began during the COVID-19 pandemic, forcing our team to pivot to virtual interviews. Thus, we were no longer able to interview people in person in their homes to understand their recovery in the context of their daily lives. As part of the pivot to online data collection, we wondered about the potential value of photovoice in facilitating pieces that were missing from the original home visit plan for data collection, including participant self-expression and self-reflection; articulation of aspects of critical illness recovery of priority to survivors; and the generation of engaging portrayals not available elsewhere. One of our patient partners (AD), who had extensive experience with photovoice, saw the potential of group photovoice with participant dialogue and discussion to garner additional insights into survivor perspectives of critical illness recovery. We also saw the innovative potential of web-based group photovoice to enhance accessibility for survivors across diverse locations and for those facing challenges with mobility and travel, which is common among ICU survivors. We did not know whether a web-based group photovoice would be feasible and what the impact of participation would be on study participants; therefore, the current pilot study was planned.

This pilot study aimed to (1) describe ICU survivor recovery after hospital discharge depicted through survivor-generated photographs and discussions about their photographs, (2) assess the feasibility of recruiting and retaining participants in a web-based group photovoice focused on ICU recovery, and (3) describe the impact of study participation.

## Methods

### Study Setting, Team, Design, and Methodology

This research was conducted in British Columbia, Canada, where publicly funded universal health care is provided to all residents. This patient-oriented research was co-led by a patient partner (AD) who had lived experience of surviving a critical illness and was involved, not as a research participant, but as a research team member from study conceptualization to knowledge dissemination. Our team collectively had clinical expertise in critical care, research expertise in survivorship, qualitative methodologies, and arts-based methods, including photovoice.

Methodologically, this pilot study emphasized group and individual reflections and discussions of participant-generated photographs, participant and researcher interpretations, and the cocreation of a virtual exhibit, although the details of the virtual exhibit are not described in detail here. We integrated the photovoice methodology with a qualitative interpretive description [16] approach when conceptualizing this study and collecting and analyzing data. Interpretive description is designed to meet the knowledge needs of applied health research and was selected for its ability to generate evidence that is relevant for clinical implications [16]. Photovoice and interpretive description share a commitment to understanding complex human experiences, particularly in health and social contexts, while remaining grounded in real-world applicability rather than generating abstract theory. Both allow for methodological flexibility and adaptation based on context, with an emphasis on respectful, meaningful representation of participants’ experiences.

We combined photovoice and interpretive description methodologies to leverage photography as a means of gaining unique, survivor-led perspectives, and interpretive description with an eye to clinical application. In contrast to conventional photovoice alone, the use of interpretive description shifted analysis and interpretation beyond participants’ experiences as revealed in their photographs, allowing for their interpretation of those experiences as well. Thus, rather than relying on formal analysis of visual data, a common analytic approach in applied health photovoice research, we centered participants’ critical reflections as an interpretive mechanism [12]. While photovoice has commonly been anchored in social justice and empowerment research with the intention of promoting community action, this study’s adaptation of the method aligned more closely with the desire to generate practice-relevant insights and participant-informed evidence to improve care. Thus, our approach became a means of engaging participants to produce evidence that privileged and affirmed their insights and fostered a meaningful and valuable research process for participants.

### Participants and Recruitment

We included participants who had received care within the past 5 years in a Canadian ICU, were admitted to the ICU for a minimum of 48 hours, were aged 19 years or older, spoke English, and could provide informed consent. Exclusions included individuals with neurological diagnoses (brain trauma and intracerebral bleed), who had undergone cardiac surgery or organ transplantation, or who were receiving palliative care. We recruited participants from late fall of 2022 to early winter of 2023 by distributing electronic study posters via e-newsletters and shared on the websites of several organizations that distribute information to patient audiences. The posters were promoted on social media platforms, including X (formerly Twitter; X Corp), LinkedIn, and various Facebook (Meta Platforms, Inc) groups. We used snowball sampling, where participants were invited to share study details with other potentially interested individuals. We have experienced tremendous success recruiting study participants using these strategies for previous studies. Prospective participants who expressed interest by contacting this study’s team by telephone or email were screened for eligibility, provided study information, and had the opportunity to seek clarifications or ask questions regarding their participation. All participants provided electronic informed consent.

Our target sample size was 4-6 participants, which was determined based on this study’s feasibility aim of having a sufficient number of participants for a group photovoice that would enable robust participant interactions. This feasibility target was informed by the research team members’ experiences of conducting photovoice research. A total of 5 individuals participated in this study, and although the sample size was relatively small, we were struck by the high information power in our dataset. Information power is an approach to determining sample size distinct from data saturation. Information power suggests that the more information the sample holds for this study, the fewer participants are needed [17]. We considered the sample to have high information power due to significant variation in participant characteristics (eg, age, medical characteristics, and time since critical illness) and the diverse experiences they described, as well as the richness of their individual interview accounts and the high quality of dialogue during group photovoice discussion sessions.

### Group Photovoice Discussion Sessions

In preparation for the group sessions, the patient-partner co-lead (AD) conducted one-on-one virtual meetings with each participant to provide them with an opportunity to recount their experiences with critical illness and establish a shared understanding and connection with the facilitator. During these one-on-one virtual meetings, AD shared with each participant that she had lived experience as an ICU survivor. Subsequently, the group photovoice discussion sessions included five 2-hour weekly virtual sessions via Zoom (Zoom Communications, Inc), facilitated by the project co-leads (AFH and AD). The discussion sessions occurred from January to February 2023. AFH took field notes following each session, capturing overall impressions of common themes and noting unique aspects of each participant’s experiences and perspectives.

The first session served as an introductory session, whereas the subsequent sessions involved participants each presenting their photos and discussing them. Each session included a brief icebreaker and overview of the session topic, as well as a wrap-up activity and review of instructions for the next session. Participants were invited to take digital photographs with a device of their choice and upload the images to the University of British Columbia’s secure version of OneDrive (Microsoft OneDrive, 2007). A photo reflection form (Multimedia Appendix 1) was emailed to participants to assist in reflecting on their photos before each discussion session, though these were not submitted to the research team.

In group sessions 2 to 5, each participant shared and discussed 2 of their photos with the group, pertaining to distinct themes assigned for each week: going home from the hospital, organizing life and using technology, mental health, and reorienting life. These themes were informed by our in-progress complementary qualitative study investigating critical illness recovery at home and drivers of hospital readmission. See Textbox 1 for an overview of the group photovoice sessions. The group cofacilitators used a discussion guide for each week’s session to ensure each participant had the opportunity to respond to the participant sharing their photo and to ensure all participants were able to share their photos in the 2 hours.

**Group photovoice content.**

**Presession**

- Virtual meeting with patient partner cofacilitator

**Session 1**

- Introduction to photovoice and photography

**Session 2**

- Systems
  What was it like to transition from hospital to home?
  What were challenges and facilitators to this transition? 

**Session 3**

- Organization/technology
  What did you do to organize and adapt your life in your home, at first and/or ongoing?
  What did you and your family need when you're at home, at first and/or ongoing? 

**Session 4**

- Emotional/mental health
  What was your emotional and mental health like after your critical illness and intensive care unit (ICU) experience?
  What are the lingering emotional and mental health effects of your critical illness and ICU experience?

**Session 5**

- Reorienting life: what’s important to understand?
  Where are you at now with your life?
  How has your life and ideas about the future changed?
  What is important for others to understand about life after a critical illness?

### Data Collection

The group photo discussion sessions were video recorded, transcribed verbatim using the machine transcription service Temi, accuracy checked, and digital photos were then embedded into the corresponding sections of the transcripts. After completing all 5 photo discussion sessions, 1 team member (KL) conducted one-on-one, semistructured interviews with each participant virtually via Zoom. We used a semistructured guide (Multimedia Appendix 1) that prompted participants to describe their experiences and perspectives of critical illness recovery and the impact of participating in the group photovoice sessions. Each individual interview lasted 45-60 minutes**.**

### Analytic Approach

Guided by interpretive description, we took an inductive thematic approach to the analysis of transcripts, aiming for interpretation rather than pure description and with an eye to clinical relevance. We relied on participants’ critical reflections of the photos generated and shared during the group photo sessions and individual interviews, rather than the research team explicitly analyzing the photographs. We used data management software NVivo (version 12; Lumivero, Inc) and conceptual diagramming software Miro throughout the analysis. Several study team members independently reviewed the transcripts to become familiar with the data. One team member (RH) read the transcripts multiple times and used an inductive coding approach to generate initial codes that represented initial patterns, ideas, and diversity related to the research objectives. Through research team dialogue and discussion, these initial inductive codes were grouped and regrouped to create a coding frame that was then applied to all transcript data. Coded data were then extracted and inductively analyzed to identify the main themes and ideas within each theme and to determine an organizing structure for presenting the findings. This involved diagramming to visualize the themes and the use of constant comparative methods to compare and contrast within and across participants, moving from descriptive to more interpretive analysis.

Research team members discussed and iteratively refined the evolving analysis during biweekly meetings. These group meetings were also an opportunity for collective reflexivity, enabling our research team members, who brought varied experiential, disciplinary, and methodological backgrounds, including a patient partner with experience of ICU survivorship, to reflect on our positionalities, assumptions, and biases, and how we were influencing the data collection, analysis, and interpretation. Through these reflective dialogues, we considered various contextual influences and deepened and clarified emerging insights. These reflexive engagements informed our ongoing analysis and refinement of our interpretations. This study’s findings are reported following the COREQ (Consolidated Criteria for Reporting Qualitative Research) guidelines and checklist [18].

We assessed the feasibility of recruiting and retaining participants in this pilot study by evaluating whether we could recruit 4-6 individuals and whether they would complete all group photovoice sessions and the postsession individual interviews.

### Ethical Considerations

This study protocol was approved by the University of British Columbia Behavioural Research Ethics Board (H22-02507). After eligibility screening, a REDCap (Research Electronic Data Capture; Vanderbilt University) link containing an e-consent form was emailed to participants, and all participants provided electronic informed consent. Consent was also reaffirmed at the beginning of each one-on-one interview. Participants each received CAD $275 (a currency exchange rate of CAD $1=US $0.73 was applicable) in e-gift card honoraria (CAD $50 for each discussion session and CAD $25 for the one-on-one interview) and were compensated after each session or interview. We attest to maintaining the privacy and confidentiality of research participants’ data and/or identity. The data were deidentified.

## Results

### Overview

A total of 4 women and 1 man participated, with a mean age of 48.6 (SD 16.8, range 22-64) years, with the majority identifying as self-reported European background (4/5), college or university-educated (5/5), married or common law (4/5), and living in a large city (4/5). Participants were admitted for sepsis (2/5), bradycardia (1/5), pulmonary embolus (1/5), or pneumonia (1/5), with the majority having been in the ICU <10 days (4/5). Before critical illness, the majority were employed (4/5); after the critical illness, a minority were employed (2/5), and the majority did not have difficulties living on their total household income either before or after their critical illness (4/5). Participants reported that at the time of this study, they had no problems with their physical activity (2/5), mild impairment in their level of physical activity (2/5), or some change in their level of physical activity such that they required bedrest less than 50% of waking hours (1/5).

The aspects of critical illness recovery that featured prominently in group photo discussions, survivor-generated photos, and individual interviews included (1) protracted physical recovery; (2) profound psychosocial challenges encompassing fear of the future, emotional turmoil, shifting self-perception, and changes in family dynamics and feelings of disconnection; (3) discrepancies in health needs and support offered; and (4) need for perseverance and resilience. The web-based group format was feasible and characterized by survivors as an opportunity to make sense of and share their experiences, feel heard, validated, and supported by others, and learn from their peers.

### Protracted Physical Recovery

Through participant-generated photos and commentary, the survivors portrayed the harrowing and physically demanding journey of recovery from critical illness at home. Many recounted the shocking realization of their profound physical decline, particularly the severe loss of muscle strength, especially after extended ICU and hospital stays. The extent of their impairment was starkly highlighted by 1 survivor’s recollection of their inability to perform even basic daily tasks, such as preparing and eating meals, drastically diminishing their quality of life. Others characterized their physical recovery as “long and arduous.” Despite making significant progress in the months and even years following their critical illness, some continued to grapple with persistent aftereffects, such as impaired physical functioning, chronic pain, and breathlessness, with one expressing deep concern that these lingering health issues would “always be a part of [their] life,” fearing she would “hurt like this forever.”

**Figure 1:**
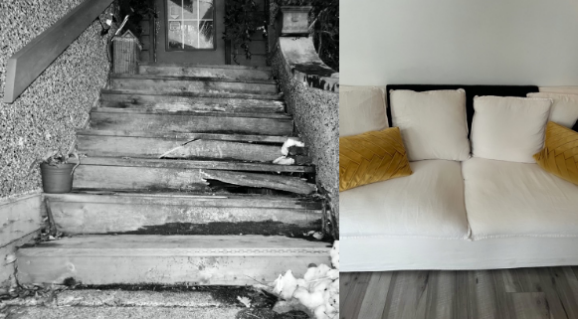
Protracted physical recovery; (left) “inviting resilience inside” illustrated a tangible barrier that also symbolized 1 survivor’s psychological challenges. (Right) “ground zero” showed another participant’s couch, the central hub where they spent their days.

Given the severity and persistent physical symptoms and impairments, survivors described their physical home environment as fraught with unexpected obstacles to their recovery. For example, 1 survivor’s photo of the stairs leading to their entryway vividly illustrated a tangible physical barrier that also symbolized the profound and related psychological challenges they faced (Figure 1, left). They described the stairs as looking unsafe, and given their physical condition, they doubted their ability to fix them, feeling daunted and overwhelmed with tasks once easily performed. Obstacles such as these stairs not only served as physical barriers, but also as stark evidence of a frightening and arduous recovery. Other survivors captured images representing the significant and sometimes extensive adaptations required for their recovery, including modifications to couches, bathtubs, toilets, and living and sleeping arrangements. Yet, obstacles in the home could not always be overcome, as depicted by a photo titled “ground zero” (Figure 1, right) of a survivor’s couch that was the central hub where they spent their days. For all survivors, the initial recovery at home appeared to be marked by seemingly insurmountable obstacles that demanded immense patience and time to overcome.

### Profound Psychosocial Challenges

#### Overview

The survivors’ photos and commentaries highlighted the profound and intense emotions they endured while recovering at home, including fear of the future, emotional turmoil, shifting self-perception, and changes in family dynamics and feelings of disconnection.

#### Fear of the Future

**Figure 2:**
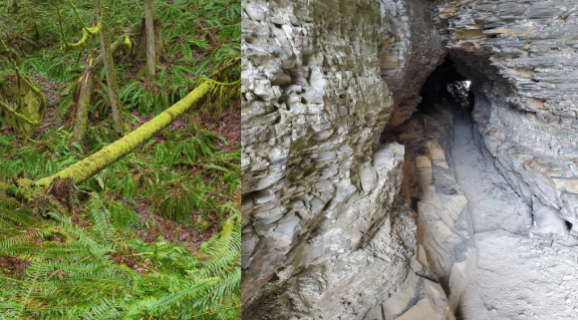
Fear of the future; (left) “fear of the future” represented the potential dangers 1 participant feared when transitioning from the hospital to home. (Right) “facing uncertainty” depicted another survivor’s feelings of uncertainty and unpredictability in their recovery trajectory.

Following their hospitalization, the survivors described the uncertainty and fear when initially coming home and then beyond. For some, leaving the safety of the hospital left them apprehensive and fearful about how they would cope at home, the support they would need, and what the future would entail. A photo of a moss-covered log (Figure 2, left) represented the potential dangers 1 survivor feared when “crossing over” from the hospital to home and not knowing what the future held.

Considering all they had endured, along with the extent and severity of ongoing health issues, the survivors questioned whether they would ever fully recover physically and emotionally and feared an unpredictable future. When discussing their photo (Figure 2, right) titled “facing uncertainty,” 1 survivor reflected on not “know[ing] how you were going to come back from that.” Survivors described their future physical health as uncertain and fragile, likened to an unclear “road ahead,” marked by discouragement, fear, and dread of incomplete recovery or further health setbacks. Moreover, reflecting on their perceived fragile health, survivors worried about their heightened risk for a recurrence of their initial illness that would land them back in the hospital, if not the ICU, as 1 person shared:

That's one of the post long haul symptoms I've had, is living with that fear of getting, for my case, getting sick again and ending up back in hospital and experiencing that all over again.

Participant 3

#### Emotional Turmoil

**Figure 3:**
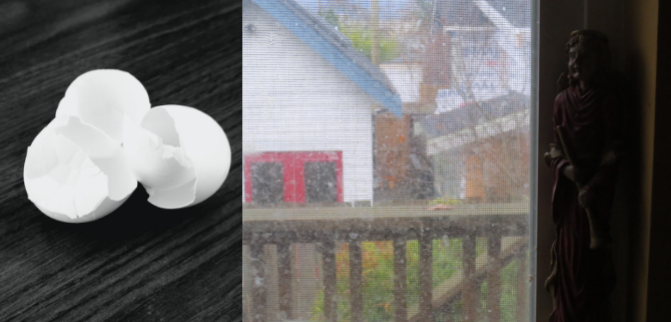
Emotional turmoil; (left) “fragile reflections: cracked resilience” illustrated 1 survivor’s sense of feeling physically and emotionally fragile. (Right) “silent retreat within” represented another participant’s desire to escape their feelings.

The surge of profound feelings—having survived a life-threatening illness, transitioning from the hospital to home, experiencing uncertainty, fear, anxiety, depression, and profound loneliness—culminated in what 1 survivor characterized as “emotional turmoil.” A survivor’s photo of cracked eggs (Figure 3, left) symbolized their sense of feeling physically and emotionally “shattered and fragile,” and overwhelmed by the extent of recovery they were facing. Another survivor described the emotional volatility and struggles they experienced after returning home as “feeling so vulnerable and just crushed,” with their “emotions all over the place” (Participant 4). This emotional fragility was especially pronounced for another survivor who wished to avoid appearing vulnerable in front of others and sought invisibility during these moments. When overwhelmed by their illness or feeling like a burden to their family, they described wanting to escape their feelings and “hide in the shadows,” like the hidden angel figurine depicted in their photo (Figure 3, right).

The profound emotional turmoil seemed to be exacerbated by the significant physical challenges the survivors faced. Accustomed to independence before their critical illness, being forced to rely on others for simple tasks at home contributed to them feeling like they were sick and incapable, heightening their sense of vulnerability even with their close family members. For example, 1 survivor shared the discomfort of needing their mother’s assistance with showering, which left them feeling extremely embarrassed during and after those moments. While another survivor spoke of their gratitude for the deep love conveyed through their son’s assistance around the home, the heavy reliance on others for daily tasks also instilled a sense of vulnerability and even shame and guilt. Transitioning from being independent and active before critical illness to relying on others, even close, trusted, and loving family members, was a disorienting change that resulted in emotional turmoil.

#### Shifting Self-Perception

**Figure 4:**
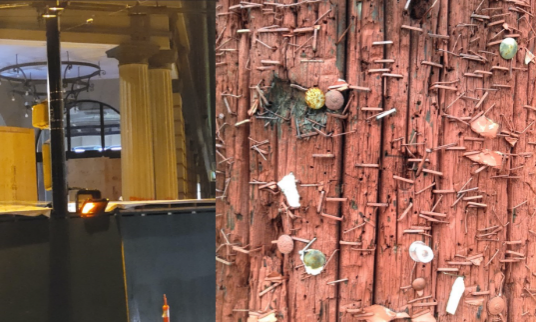
Shifting self-perception: (left) “fallen light, rising resilience, and embracing self-worth” illustrated 1 survivor’s sense of appearing broken on the outside but still functional and strong within. (Right) “transforming reflections: resilience in change” depicted another participant’s shifts in self-perception through unhealthy coping mechanisms.

Upon returning home, the survivors conveyed feeling as though everything had changed: their homes, themselves, and how others perceived them. One survivor indicated that hospitalization “created distance and disconnect from who I was before and from others, making the world feel out of reach.” Several survivors linked their lowered self-worth to “dehumanizing” hospital experiences, such as overhearing ICU clinicians’ condescending comments about themselves and other patients, which eroded dignity, lingering for months and even years. Shifts in self-perception also appeared to be closely tied to physical and functional impairments. One survivor’s photograph of a broken pedestrian light (Figure 4, left) symbolized their sense of “lower value,” and the struggle to “prove my competence,” reflecting feelings of powerlessness and visible brokenness.

The survivors commonly described shifts in their self-perception “mentally and physically,” and the need to rebuild their self-identity and navigate insecurities through environmental- and self-modifications. Seeing their much-altered reflections in the mirror, 1 survivor recalled removing all mirrors in the house, making drastic wardrobe changes, and cutting their own hair, in what they indicated were “reckless attempts to feel a sense of self.” Environmental triggers, such as sounds, smells, and lighting, commonly continued to trigger painful memories and feelings about themselves. Some survivors described feeling lost while trying to navigate their shifts in self-perception and doing so through unhealthy coping mechanisms, as depicted by 1 survivor’s photograph of staples on a telephone pole (Figure 4, right):

The remaining staples and tacks remind me of the surgeries I’ve had, leaving staples in my lungs and metal twist ties in my chest. At one point, I just thought, what is the use? I'm doomed anyway. So, I started smoking three years after I'd had heart surgery.

Participant 2

With time, some described cultivating awareness of what they needed to rebuild their self-worth and self-esteem. One survivor reflected on learning “to surrender and just see things differently…find happiness within [myself]…without keeping friendships or situations that don’t serve [me] anymore” (Participant 5).

#### Changes in Family Dynamics and Feelings of Disconnection

After having relied so heavily on hospital staff, the survivors described longing for independence in their daily lives during their recovery. Yet, their impaired health and functioning meant they were reliant on their family caregivers in ways they had not been before, and unable to resume their roles in the household, such as cooking, shopping, and caring for others. This contributed to shifts in how they perceived their roles in the family and appeared to result in changes in family dynamics. While the survivors expressed gratitude for the love, care, and support of family members, they commonly indicated feeling overly reliant on others and guilt for burdening them.

The survivors’ photos and narrations also conveyed a profound sense of emotional disconnect from their family members, who the survivors thought could not fully relate to their life-threatening ordeal and subsequent challenges. One survivor reflected, “I mean, unless you've really experienced that and you've been through that, I don't think people really do understand or can understand.” They perceived that their families were ready to move on from the critical illness experience. Yet, the survivors continued to grapple with the physical and emotional repercussions. Many felt as if their lives had come to a standstill while everyone else’s continued, making it difficult to feel connected with friends and family and exacerbating feelings of being misunderstood and lonely.

This sense of alienation left survivors perceiving themselves to be on a “different level, different frequency, different growth” than their family members. Despite yearning for validation and understanding, several survivors described being unable to confide in their family members and being compelled to protect them, even at the expense of their own emotional well-being. Consequently, 1 survivor created a photo of themself smiling but overlayed blue tears on their cheeks to depict their masking of emotions, leaving them feeling utterly alone with their physical and emotional pain. They explained,

Despite experiencing vulnerability and emotional turmoil, I felt compelled to protect those around me and prioritize their well-being. My family, unaware of the severity of my condition, struggled to cope, and I was preoccupied with their emotions rather than addressing my own. I faced anxiety, panic attacks, and physical pain alone, unable to openly express my struggles… I felt like for everybody else I had to put on that mask, or slap on that smile, or put on that happy face and just pretend that everything was fine… I was trying not to make everybody else worry.

Participant 4

Another survivor recounted negative interactions with their family members, who conveyed limited empathy and understanding of the suffering they continued to endure, indicating they had “no one really in [their] immediate environment that [they] could truly empathize with or that could support [them].” This survivor recalled their family’s disappointment in their slow recovery, leaving them feeling rejected, invalidated, and ashamed, with no one to turn to for practical or emotional support, deepening their sadness and isolation in recovery.

### Need for Perseverance and Resilience

**Figure 5:**
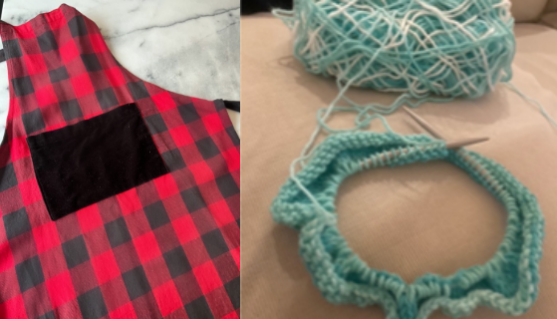
The need for perseverance and resilience; (left) “hiatus” represented 1 survivor’s resumption of previous responsibilities that were ultimately too much to manage. (Right) “stitches of resilience” depicted another participant’s process of building resilience.

Through their photos and narratives, the survivors depicted the immense difficulty of confronting numerous challenges while recovering at home. Still ongoing, this process was a bumpy road, a journey fraught with setbacks and obstacles. Survivors described making tremendous efforts to heal and resume their family, work, and social roles and responsibilities. However, with no advice on how to do so and uncertainty about what they could do, this was not always straightforward, and setbacks were commonly described. For example, 1 survivor’s photo of their apron (Figure 5, left) represented their resumption of previous responsibilities that were ultimately too much to manage, leading to a pivotal moment in their recovery journey:

After a period of progress and resuming my daily responsibilities, exhaustion and emotions caught up with me, leading to a traumatic panic attack three months later. Recognizing the need for space and clarity, I took a trip…. This time away allowed me to reflect, reassess my priorities, and make important changes in my life…. Hitting rock bottom physically and mentally ultimately led me to emerge stronger.

Participant 5

With time, all the survivors indicated that their mental health improved, though this took intentional effort. For 1 individual, their beloved cat provided a “sense of normalcy and unconditional love,” providing constant companionship and “serving as a source of comfort during times when I felt misunderstood or alone.” Mental health counseling was helpful for 2 survivors, while others found ways to help make sense of their experiences, express and manage their emotions, and set future goals. Useful tools included journaling, vision boards, and reading about others’ experiences and sharing their own experiences online.

The survivors also shared their journey of recovery as encompassing a shift in their self-perception, personal growth, and a transformed outlook on life. Some described learning to embrace their current circumstances by reframing their fears and obstacles as opportunities for mental and physical self-growth, as highlighted by 1 survivor’s comment that their “fears actually could be the best thing that [ever happened].” Others portrayed self-acceptance of the “new versions” of themselves as key to embracing their current circumstances, reflecting that “it's okay to accept that you may never be that former self again.”

Some survivors appeared to draw strength from the knowledge that they survived a life-threatening illness and continued to persevere through recovery. Acceptance, positivity, and resilience were fostered by cultivating an attitude that they were a “work in progress” and that “this too shall pass.” The survivors described embracing the imperfection in recovery, emphasizing the significance of trying to have a “humbling outlook and gratitude in life.” One survivor symbolized their ongoing healing and building of resilience with a photo of a hat they were knitting (Figure 5, right), indicating that “just like this unfinished hat, I will continue to work on myself, knowing one day I will reach a point of completion and resolution.”

### Unmet Health Needs

Throughout group discussions and individual interviews, the survivors remarked on significant unmet health needs due to insufficient follow-up care after being discharged from the hospital. They emphasized the desire for a mentorship or support group or a point of contact for help when leaving the hospital to help address the “massive gaps in [the] healthcare system.” They also suggested ICU diaries and journals to help survivors and their family members fill memory gaps. The survivors commonly indicated that the lack of mental health support severely and negatively impacted their recovery:

If there's anything the system really needs to improve on it’s the treatment of patients and also to focus on the mental aspect of being hospitalized because we felt like once we were discharged, they kind of just forgot about us and they never really followed up.

Participant 1

**Figure 6:**
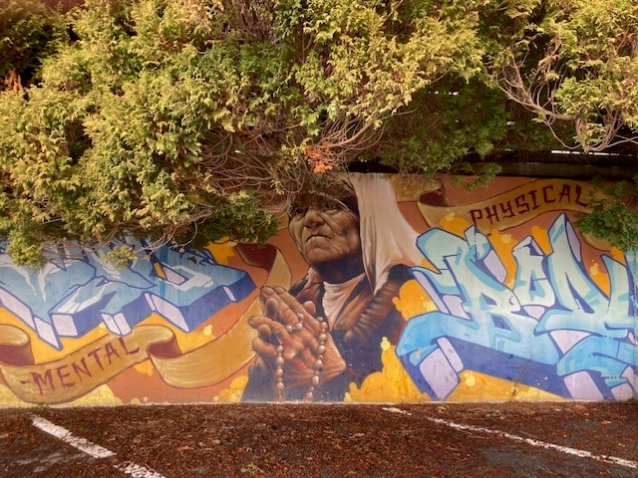
Unmet health needs; “the art of noticing” illustrated the link between mental and physical health in recovery.

The survivors highlighted the intrinsic link between mental and physical health in their commentaries and photos (Figure 6), noting that “if you're not well mentally, you can't do well physically.” They expressed frustration with the prioritization of physical symptoms while neglecting emotional and mental health aspects.

### Feasibility of Web-Based Group Photovoice

Five participants were recruited, all of whom completed all group photovoice discussion sessions, submitting a total of 42 photographs, and completing a one-on-one interview, thus suggesting the feasibility of this study’s protocol. However, given that there are no dedicated follow-up clinics, aftercare programs, or recovery initiatives in the province to recruit through, reaching eligible participants proved challenging, which prolonged the process of finding participants available and willing to commit to the project. A few months following the completion of the group sessions, all survivors became patient partners to pilot the development of a website [19] to disseminate their curated photos.

### Impact of Web-Based Group Photovoice

#### Overview

Although the web-based group photovoice was not initially intended to have a therapeutic impact, survivors overwhelmingly reported immense benefits from participating.

#### Power of Images: Making Sense and Sharing

The group photovoice experience enabled survivors to revisit and reinterpret their ICU experiences and recovery at home. Participants reported that the structured photograph assignments prompted self-reflection, helping them make sense of all they had been through. This introspective journey was therapeutic and empowering, reinforcing their identities as resilient survivors*.* The group setting also offered a unique platform for sharing and interpreting their photographs, with survivors struck by the power of photography to convey emotion and understanding meaning. Centering the photographs during sharing sessions provided an opportunity for individual expression and communal connection, as participants found resonance and validation in the images and explanations of others:

Some [photos] were very powerful images and spoke very profoundly to my own journey…. We looked at [someone else’s] picture and thought “[I can] hardly wait to hear the story behind the picture.” Each one of them always seemed to hit the mark once you got the explanation.

Participant 3

The process of sharing and interpreting photographs within the group context required a high degree of openness, which participants also found to be “humbling” and a “privilege.”

#### Feeling Heard, Validated, and Supported by Others

The survivors described discovering a powerful sense of community, exchanging advice and “words of wisdom” with others who had also endured critical illness. This newfound connection was characterized as providing strength and validation, helping them realize they were not alone in their struggles. Survivors expressed a sense of being profoundly heard and understood in a way they had not before, and “like nobody else could.” One survivor’s description conveyed the deep emotional connections, the impact of empathy, and the healing that was facilitated by the group discussions.

To be able to talk about it so freely and to know that people can understand and empathize was so comforting. And I think I really needed that. I don't think I really realized how validating it would be to talk about it with a group where they've also experienced the same treatment…. It makes me quite emotional to think that... people [I] have never met, really were able to make me feel so whole and so strong again.

Participant 1

Further, the survivors highlighted that the development of group cohesiveness was rapid and profound. They reported an immediate sense of belonging and genuine care, contributing to a supportive and safe environment. This nurturing atmosphere was conveyed as crucial in enabling the survivors to share sensitive personal experiences and receive empathetic feedback. Several remarked on the transformative power of group photovoice, describing it as a “genuine expression of experience,” “part of a healing process that [I] didn’t even know existed,” “the missing link in [my] recovery,” and a potential “form of therapy.”

#### Learning From Their Peers

Survivors expressed that their fellow participants helped them learn coping skills and ways to adjust to their current situation. One survivor commented on reorienting their outlook to a more “positive approach” based on what another survivor had exuded. The survivors appreciated “all these different views, points of view, or at least expressions of experience.” The exposure to the group’s diversity in age demographics, life experience, and time duration from ICU discharge was seen as a major strength since it allowed participants to learn from the vast range of experiences. Being able “to provide that sense of hope and encouragement to those that aren't quite there yet” was described as healing in itself. Empowered by the empathy and compassion of fellow participants, survivors noted they felt that they could lower their protective guard, embrace hope for their ongoing recovery, and offer comfort and support to others on a similar journey.

## Discussion

### Principal Findings

Our findings highlight aspects considered by critical illness to be central to recovery, namely, immense physical and emotional health challenges along with shifting self-perception, changes in family dynamics, and feelings of disconnection. Despite the enormity of health needs, these were not accompanied by access to appropriate health care services or support. Recovery was further depicted as requiring substantial perseverance, but also contributing to resilience and strength. The group photovoice web-based format was feasible and perceived by survivors to be an opportunity to make sense of and share their experiences, feel supported by others, and learn from their peers.

### Comparison to Prior Work

Our findings align with the robust literature describing the persisting physical sequelae and functional impairment common post-ICU [20,21] and extend knowledge of how the home environment can be fraught with obstacles to recovery. These obstacles were unexpected and required marked effort from survivors and, oftentimes, their family members to make the necessary adaptations. Our findings suggest that functional impairments are not solely inherent to individuals; rather, the home environment plays an under-recognized and modifiable role in influencing recovery. Our photovoice approach enabled participants to convey the contexts shaping their everyday realities experienced during recovery. Given that ours was a small pilot study, additional research is warranted to understand the extent and nature of obstacles to recovery commonly encountered in the home, adaptations to support survivors in their activities of daily living, and the role that occupational therapy and home assessments could play.

Emotional challenges of fear of the future, uncertainty, vulnerability, emotional turmoil, guilt, loneliness, and social disconnect figured prominently in our study. These appeared to encompass a much broader array of psychological sequelae than the anxiety, depression, and posttraumatic stress that are most often described in the literature [3,4]. Further, the emotions highlighted by participants through their photographs not only stemmed from physical and cognitive sequelae but were entwined with newfound dependence on others, which was couched in shame, and changes in family dynamics. These relational dimensions of recovery were uniquely conveyed through participants’ photographs and commentary. Further exploration of the full scope of emotional challenges may reveal targets for innovative psychosocial interventions, such as those found in survivorship research for other illnesses. For example, in the last decade, fear of cancer recurrence has emerged as a problematic long-term issue for survivors of cancer, with promising treatment options being investigated [22]. Similarly, while a small but growing body of literature suggests that survivors of critical illness may experience changes in personal identity and outlook [3,23], the participants in our study conveyed the importance of their experiences of shifting self-perception. Pursuing this line of inquiry in future research might inform the development or adaptation of interventions or approaches targeting identity that show promise elsewhere, such as narrative therapy [24-26].

An important finding from this pilot study that warrants further inquiry was the participants’ photographs and narratives of perseverance and building of strength and resilience, which may be akin to posttraumatic growth. By inviting participants to express what was most meaningful in their experiences, the photovoice approach shifted the findings from a focus solely on challenges commonly described in the literature to also include aspects of recovery where participants found meaning, ways of coping, and strength. Posttraumatic growth is the experience of positive psychological change that can result from the struggle with life crises and trauma, and can manifest in a variety of ways, including an increased appreciation for life, more meaningful interpersonal relationships, an increased sense of personal strength, changed priorities, and a richer existential and spiritual life [27]. Jones et al [28] made a solid argument for the relevance of the concept for survivors of critical illness, noting parallels to other populations who experience trauma. They also proposed strategies and mechanisms that could promote posttraumatic growth during and after ICU stays and suggested avenues for future research including, integrating assessments of posttraumatic growth into ICU aftercare and self-help resources, exploring which domains and trajectories of posttraumatic growth are unique to ICU survivors, determining which interventions could foster posttraumatic growth after critical illness, and identifying effective methods to disseminate and implement this knowledge among providers to encourage posttraumatic growth in patients [28].

Our findings resonate with scholarship on illness narratives, especially the ways that survivors of critical illness described disrupted embodiment and self-identity. Frank [29] proposed that illness narratives often reflect altered experiences of being in one’s body, the search for meaning, self-identity reconstruction, and repositioning of relationships. Our participants also expressed uncertainty, shame, and vulnerability that appeared to reflect existential disruptions associated with illness and a disintegration of the taken-for-granted body and a reorientation in sense of self [30]. The shame associated with newfound dependence and altered family dynamics also aligns with dignity-focused scholarship, which emphasizes the psychosocial and relational dimensions of suffering and identity erosion during serious illnesses [31]. By surfacing these layered embodied, emotional, and relational dimensions through photographs and related narratives, our study uniquely situates post-ICU recovery as not only medical or functional but also as an embodied, existential, and socially mediated experience. As a medium of expression, photovoice helped convey the recovery experience from the vantage of survivors.

The participant-generated photographs and commentary in our study served as distinctive and authentic portrayals of aspects of critical illness recovery that were important to participants. Recognizing the value of these portrayals to provide insight and validation, our team piloted the codevelopment with all study participants of a virtual gallery as a knowledge translation strategy [19]. Among other populations, photovoice exhibits can be a powerful means of confronting gaps between what researchers and policymakers assume people experience and what people show that they experience and need [32]. By creating space for participants to convey their own ideas and experiences, photovoice can reinforce people’s right to be seen and heard as they choose [33]. This is particularly relevant for people who have survived critical illness, during which vulnerability and loss of control can be marked. Photovoice exhibits can further serve as a means of moving findings outside traditional academic settings, perhaps to be more appealing and accessible to public audiences, and a bridge to action and change at individual, interpersonal, community, institutional, and policy levels [32,34]. Further research examining the impact of photo exhibits in the context of critical illness recovery could provide novel means of raising awareness and inspiring change.

### Strengths and Limitations

Our piloting of web-based group photovoice suggested that this approach was not only feasible but was also impactful. The therapeutic benefits and enhancement of social ties apparent in our study seem akin to benefits found in arts-based research, including photovoice, reported in other populations [15,33,35]. The use of group photovoice and even other arts-based methods ought to be studied further as a complement to existing interventions, such as peer support groups. Emerging evidence suggests that peer support among survivors of critical illness can be beneficial, with many of the mechanisms similar to those we witnessed in our group photovoice study. By sharing experiences through peer support groups, survivors have reported diminished anxiety and concerns, enhanced motivation and hope, feeling validated, gaining a greater understanding of their ICU illness and care trajectory, and experiencing a sense of purpose in assisting others [36,37]. Our group photovoice was facilitated by a patient partner who was an ICU survivor themselves. This approach seemed to foster trust and rapport, with participants coming to the first workshop session appearing comfortable sharing sensitive experiences. This peer connection may have enhanced the relevance and depth of the data, as well as the participants’ sense of mutual support. However, this peer facilitation may have also introduced potential biases, such as assumptions of shared understanding and a failure to fully explore aspects not aligning with those of the facilitator. Further, social desirability may have influenced participants such that they shaped their narratives and photographs to align with shared experiences or the facilitator’s expectations.

While our findings are promising, it is important to note that this was a pilot study with a small, self-selecting sample. Future research is required to assess the scalability and potential integration of group photovoice into clinical programs or existing care structures. Further, a key consideration in photovoice, like other arts-based methods, is the potential for exclusion. Collective and creative formats might not appeal to all individuals, particularly those less comfortable with expressive modalities or group-based activities. As observed in our sample, participation skewed toward self-reported European background, more educated individuals, living with a family member, and in an urban area, suggesting a need to consider means of promoting accessibility, equity, and cultural appropriateness in recruitment efforts and future offerings of group photovoice. Otherwise, future research risks contributing to health inequities. Moreover, while virtual delivery enhanced accessibility and flexibility, it also may have introduced inequities related to digital literacy, technology access, and privacy that warrant future consideration. Future research ought to attend to these structural and digital barriers in study design, recruitment, and support for participation to promote photovoice that is inclusive and representative of diverse survivor experiences.

This pilot study, conducted in Canada during the COVID-19 pandemic, involved 4 women and 1 man, most of whom had some university or college education and were financially stable. Consequently, the insights into critical illness recovery presented may not reflect the experiences of individuals in different geographic or socioeconomic contexts. The mental and physical strains of the pandemic may also have influenced the results. Additionally, the benefits observed from participating in web-based group photovoice sessions may not extend to those who did not choose to participate, or to individuals who do not see value in sharing experiences or who are not emotionally prepared for such engagement. Photovoice demands significant time and emotional investment, which many ICU survivors may not be able to afford, especially at time points in their healing when they are at their most vulnerable. While increasing access geographically, the virtual format may also limit the participation of those with minimal access to a computer or phone, possibly excluding individuals facing significant social and structural challenges.

### Future Directions

Given the insights our small pilot study has provided into lesser-explored aspects of critical illness recovery, the potential therapeutic value and ability of web-based group photovoice to foster social connectedness, and possibilities for innovative knowledge translation, future research is warranted to assess the impact of a scaled-up application.

## Appendix

Multimedia Appendix 1 The photo reflection form and the one-on-one interview guide.

## Abbreviations

COREQ: Consolidated Criteria for Reporting Qualitative Research
ICU: intensive care unit
PICS: postintensive care syndrome
REDCap: Research Electronic Data Capture
