# Supplementary material for: Web-Based Group Photovoice Through the Lens of Survivors of Critical Illness Recovery: Photovoice Qualitative Pilot Study
Source: JMIR Form Res. 2026 Jan 20;10:e66601. doi: 10.2196/66601 (PMC12869148; doi:10.2196/66601)
Supplement: Multimedia Appendix 1 [file formative_v10i1e66601_app1.docx]

**Information S1: Photo Reflection Form**

This photo reflection form is intended to assist you in thinking about your photos and what you would like to discuss during the group discussions. This form is for your use only and will not be returned to the study team.

DATE:

**Photo # 1**

Photo Title:

Please describe your photo.

What is missing from your photo?

Why do you want to share this photo?

What’s the unseen story of your photo?

How does this relate to your life?

**Photo # 2**

Photo Title:

Please describe your photo.

What is missing from your photo?

Why do you want to share this photo?

What’s the unseen story of your photo?

How does this relate to your life?

**Information S2: One-on-One Individual Interview Guide**

We are interested in the photographs you took and what you intended to convey as it relates to:

- your experiences of recovering from their critical illness at home,
- challenges that that might have contributed to declining health and hospital readmission, and
- how you successfully overcame challenges, or not, when recovering from critical illness following hospital discharge?

**Individual Photo Reflections**

I am going to display the 4 photographs you indicated best represent important aspects of your experiences. I invite you to reflect on your experiences and will ask you a few questions throughout our discussion.

1. Describe what you are conveying about your experience in this photo.
2. Is there anything you wanted to share about your experiences that you did not get a chance to talk about during the group photo sessions?
3. Which would you like to share as part of a virtual public display?

**Group Photo Discussion Sessions**

1. In thinking about the group photo discussions, what stood out as important in the photos and reflections?

- Related to system challenges
- Related to organization/technology
- Related to mental/emotional health
- Related to reorienting life
- Other

1. What do you see as some of the shared experiences of surviving a critical illness?

- Experiences of recovery
- Challenges
- Ways of overcoming challenges?

1. What do you think is an important priority in helping people recover from critical illness?

**Acceptability of the Group Sessions**

1. How did you feel throughout the group photo discussion sessions?
2. How was it for you to share your photos and reflections?
3. How did seeing the photos and hearing the reflections of others impact you?
4. What, if anything, was concerning or difficult for you throughout the process?
5. What, if anything, was beneficial for you throughout the process?
6. Would you recommend workshop participation to others who have survived a critical illness? Why or why not?
7. What changes would you recommend to the process?

**Involvement in Next Steps**

1. Who do you think would benefit from a virtual exhibit?
   1. Family/friends
   2. Other survivors of a critical illness
   3. General public
   4. Healthcare clinicians and decision makers
2. How do you feel about sharing your photos in a virtual exhibit?
3. Would you like to be involved in any planning activities for a virtual exhibit?
   1. Helping to make decisions about the virtual exhibit?
   2. Helping to refine materials (ie brief photo descriptions for your photos)?
